# Supplementary material for: The Systems Biology Research Tool: evolvable open-source software
Source: BMC Syst Biol. 2008 Jun 29;2:55. doi: 10.1186/1752-0509-2-55 (PMC2446383; doi:10.1186/1752-0509-2-55)
Supplement: Additional file 1 — SBRT Archive. An archive of the current version of the Systems Biology Research Tool. [file 1752-0509-2-55-S1.zip › sbrt-1.4.0/doc/developers_guide/api/sbrt/kernel/fba/proc/NetworkInfoProcess.html]

NetworkInfoProcess


|  |  |  |
| --- | --- | --- |
| **Overview** \| **Package** \| **Class** \| **Use** \| **Tree** \| **Deprecated** \| **Index** \| **Help** | |  |
| **PREV CLASS**   **NEXT CLASS** | **FRAMES**    **NO FRAMES**     **All Classes** |  |
| SUMMARY: NESTED \| FIELD \| CONSTR \| METHOD | DETAIL: FIELD \| CONSTR \| METHOD |  |


---


## sbrt.kernel.fba.proc Class NetworkInfoProcess

```
java.lang.Object
  sbrt.kernel.fba.proc.FbaProcess<NetworkInfoProcess.Results>
      sbrt.kernel.fba.proc.NetworkInfoProcess
```

**All Implemented Interfaces:**: KernelProcess<NetworkInfoProcess.Results>

---

``` public final class NetworkInfoProcess extends FbaProcess<NetworkInfoProcess.Results> ```

This class is used to gather basic information about a fluxome.

**Author:**
:   This class was written and documented by Jeremiah Wright
    while in the Wagner lab.

---

| **Nested Class Summary** | |
| --- | --- |
| `static class` | `NetworkInfoProcess.Results`             This class is used to represent the information gathered about a fluxome. |


**Constructor Summary**

`NetworkInfoProcess(CatalyzedFluxome fluxome)`
  
          Constructs a new flux activity experiment.


| **Method Summary** | |
| --- | --- |
| `CatalyzedFluxome` | `getFluxome()`             Returns the fluxome for which information is to be gathered. |
| `void` | `run(ResultsManager<NetworkInfoProcess.Results> resultsManager)`             Adds the information gathered about the fluxome to the provided results manager. |

**Methods inherited from class sbrt.kernel.fba.proc.FbaProcess**

`toString`

**Methods inherited from class java.lang.Object**

`clone, equals, finalize, getClass, hashCode, notify, notifyAll, wait, wait, wait`

**Constructor Detail**

### NetworkInfoProcess

```
public NetworkInfoProcess(CatalyzedFluxome fluxome)
```

:   Constructs a new flux activity experiment.

    **Parameters:**: `fluxome` - the fluxome for which information will be gathered.


**Method Detail**

### getFluxome

```
public CatalyzedFluxome getFluxome()
```

:   Returns the fluxome for which information is to be gathered.

    :   **Specified by:**: `getFluxome` in class `FbaProcess<NetworkInfoProcess.Results>`
    :   **Returns:**: the fluxome for which information is to be gathered.

---


### run

```
public void run(ResultsManager<NetworkInfoProcess.Results> resultsManager)
```

:   Adds the information gathered about the fluxome to the provided
    results manager.

    :   **Parameters:**: `resultsManager` - the results manager to which the gathered information will be added.


---


|  |  |  |
| --- | --- | --- |
| **Overview** \| **Package** \| **Class** \| **Use** \| **Tree** \| **Deprecated** \| **Index** \| **Help** | |  |
| **PREV CLASS**   **NEXT CLASS** | **FRAMES**    **NO FRAMES**     **All Classes** |  |
| SUMMARY: NESTED \| FIELD \| CONSTR \| METHOD | DETAIL: FIELD \| CONSTR \| METHOD |  |


---
